# Mediastinal Intrathymic Parathyroid Adenoma: A Case Report and Review of the Literature

**DOI:** 10.7759/cureus.42306

**Published:** 2023-07-22

**Authors:** Benjamin M Abraham, Elise Sharkey, Lily Kwatampora, Mark Ranzinger, Urs von Holzen

**Affiliations:** 1 College of Osteopathic Medicine, Marian University, Indianapolis, USA; 2 Center for Cancer Care, Goshen Health, Goshen, USA; 3 Department of Endocrinology, Goshen Health, Goshen, USA

**Keywords:** hypercalcemia, hyperparathyroid, video-assisted thoracoscopic surgery (vats), spect-ct, solitary parathyroid adenoma, ectopic parathyroid tissue, intra-thymic

## Abstract

The classic clinical vignette of primary hyperparathyroidism is well described as “bones, stones, abdominal moans, and psychiatric overtones” to reflect the effects of excess parathyroid hormone (PTH) and calcium. Most commonly, primary hyperparathyroidism is due to a functional parathyroid adenoma situated by the thyroid gland. Rarely, the primary focus of autonomously produced PTH is located ectopically within the mediastinum.

A 19-year-old Caucasian female with no relevant past medical history presented to the emergency department with tachycardia, nausea, vomiting, and a five-day history of vague, mid-abdominal pain. Initial computed tomography (CT) with contrast of the abdomen and pelvis was negative for acute findings, and she subsequently underwent biochemical screening. The patient was found to have elevated serum calcium and PTH, raising suspicion for the diagnosis of primary hyperparathyroidism. Further evaluation for a parathyroid adenoma was negative by a CT scan of the neck and thyroid ultrasound. A nuclear medicine parathyroid single-photon emission computed tomography (SPECT)/CT with technetium (Tc) 99m sestamibi found an abnormal nodular uptake within the left prevascular mediastinum suggestive of an ectopic parathyroid adenoma. A left-sided, video-assisted thoracoscopic surgery (VATS) with successful excision of the ectopic mediastinal parathyroid adenoma was performed. Surgical pathology revealed that the parathyroid adenoma was completely excised and surrounded by thymus and adipose tissue. The patient tolerated the procedure well and was discharged without further complications.

The rarity of mediastinal, intrathymic parathyroid adenomas resulted in delayed diagnosis in this patient, understandably so as errant embryogenesis does not occur commonly. Visualization with SPECT/CT and successful specimen excision by minimally invasive VATS resulted in the accurate diagnosis and ultimate cure of this patient’s primary hyperparathyroidism.

## Introduction

Primary hyperparathyroidism is the most common cause of hypercalcemia and the third most common endocrine pathology [1]. The most common cause of primary hyperparathyroidism is a single parathyroid adenoma [2].

Although primary hyperparathyroidism is most commonly caused by a single benign adenoma located in the peri-thyroid region [3], ectopic foci present a diagnostic challenge when initial localization attempts are negative. A basic awareness of normal parathyroid embryogenesis is useful in the process of clinical inspection for ectopic sites secondary to errant tissue migration. From an embryological perspective, the superior parathyroid glands are derived from the fourth pharyngeal pouches and migrate a relatively short distance. Therefore, ectopic superior parathyroid glands are most frequently found in the retropharyngeal and retroesophageal locations [4]. In contrast, the inferior parathyroid glands and the thymus are both derived from the third pharyngeal pouches, and these together travel a relatively longer distance before reaching their anatomical destination [4]. Ectopic inferior parathyroid glands are more commonly described in the literature and most commonly reside within the thyroid parenchyma, mediastinum, carotid sheath, and thyrothymic ligament [4,5]. If ectopic parathyroid glands are not properly screened during preoperative imaging, this can result in a failed parathyroid exploration [6], thereby exposing patients to unnecessary risks of surgery. Preoperative efforts to precisely locate the causative lesion optimize curative rates.

Herein, we report a case of a 19-year-old patient with a symptomatic parathyroid adenoma located in the mediastinum, encased within the thymus. During the patient’s hospital course, informed consent was obtained from the patient for the presentation of her case along with associated medical imaging.

## Case presentation

History of present illness

A 19-year-old female presented to the emergency department with tachycardia, nausea, vomiting, and a five-day history of vague, mid-abdominal pain. The patient reported two similar episodes within the previous 12 months. These episodes were separated by six months and the patient did not seek formal medical attention. She started taking several over-the-counter (OTC) supplements immediately following her second episode and presented to our emergency department three months later. Upon review of systems, the patient reported a history of weight loss, heart palpitations, abdominal pain, constipation, polyuria, polydipsia, insomnia, and easy bruising following her first episode. The patient’s family history was only positive for a maternal history of thyroid disorder and heart disease. The remainder of the patient’s social, surgical, and medical histories were unremarkable. She was alert and oriented to person, place, problem, and time with appropriate mood and affect. Besides tachycardia, there were no pertinent findings on the presenting physical examination and the patient appeared to be a well-developed, well-nourished female in no acute distress.

Upon arrival, imaging and biochemical analyses were ordered to determine the etiology of abnormal findings. Abdominal and pelvic computed tomography (CT) imaging revealed no acute findings. Extensive serology studies were performed, including a comprehensive metabolic panel (CMP), complete blood count (CBC), and hormone/mineral/vitamin diagnostics. While all other lab findings were within normal limits, the measured total calcium (13.5 mg/dL) and parathyroid hormone (PTH) (514 pg/mL) levels were elevated.

Discharge

Further in-patient imaging, including CT soft tissue neck without/with contrast and thyroid ultrasound, were both within normal limits. A one-time intramuscular dose of 50 IU of calcitonin was administered and followed with intravenous pamidronate 60 mg to reduce serum calcium levels. During her hospital stay, she was scheduled on intravenous sodium chloride 0.9% 1000 mL at 150 mL/hour and a one-time 1 gram/100 mL at 100 mL/hour magnesium bolus. She was discharged 44 hours following arrival with twice-daily magnesium oxide 400 mg tablets and twice-daily potassium chloride 20 mEq extended-release tablets and was scheduled for an urgent out-patient endocrinology consultation in the face of persistently elevated in-patient serum calcium and PTH levels.

Radiology and endocrinology evaluation

**Figure 1:**
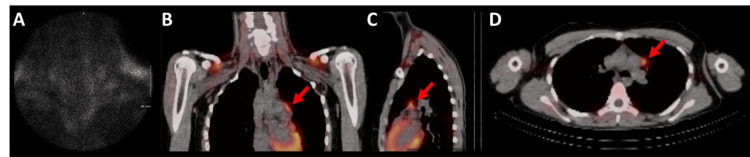
Parathyroid and thyroid scans Representative nuclear medicine studies indicating a lack of avidity in the peri-thyroid region (panel A) with a concomitant increase in metabolic activity around the prevascular mediastinal area (red arrows, panels B-D) overlayed on non-contrast CT imaging in coronal (panel B), sagittal (panel C), and axial (panel D) planes. Following intravenous (IV) administration of technetium-99m sestamibi, the thyroid (panel A) and mediastinum (panels B-D) were evaluated for increased uptake. The thyroid, demarcated by black (low activity) to white (high activity) shading (panel A), showed uptake within normal ranges. However, the mediastinum, demarcated by red (low activity) to yellow (high activity) shading, showed an unusual focus of increased uptake in the prevascular mediastinal area (red arrows, panels B-D).

The endocrinologist obtained a neck and chest parathyroid scan with single-photon emission computed tomography (SPECT)/CT with technetium (Tc) 99m sestamibi (administered intravenously). There was a lack of tracer avidity in the thyroid bed, neck, and typical parathyroid regions (Figure 1A). However, the study revealed a 17 mm x 11 mm prevascular mediastinal nodule with moderate uptake, consistent with an ectopic parathyroid adenoma (Figures 1B-1D).

**Figure 2:**
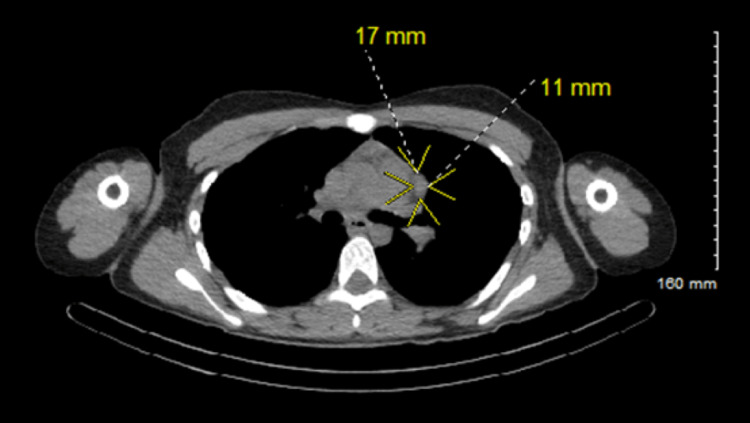
Confirmatory CT imaging of prevascular mediastinal parathyroid adenoma High-quality CT scan without contrast of the chest with isolation (yellow arrows) and orthogonal measurements based on the longest primary axis of the ovoid 17 mm x 11 mm mediastinal lesion (yellow-colored font adjacent to white-dashed line).

High-quality CT imaging without contrast of the mediastinum was utilized to better visualize the likely ectopic parathyroid adenoma. There was a demonstration of a 17 mm x 11 mm ovoid soft tissue nodule in the prevascular position along the lateral left aspect of the main pulmonary artery trunk (Figure 2).

To rule out the possibility of multiple endocrine neoplasia type 1 or 2a (MEN 1/2a), levels of adrenocorticotropic hormone (ACTH), prolactin, aldosterone, plasma renin, and cortisol were analyzed. The patient’s ACTH, prolactin, aldosterone, plasma renin, and cortisol laboratory studies were all within normal ranges, supporting that the ectopic parathyroid adenoma was not part of a syndromic presentation.

Parathyroidectomy and surgical pathology

**Figure 3:**
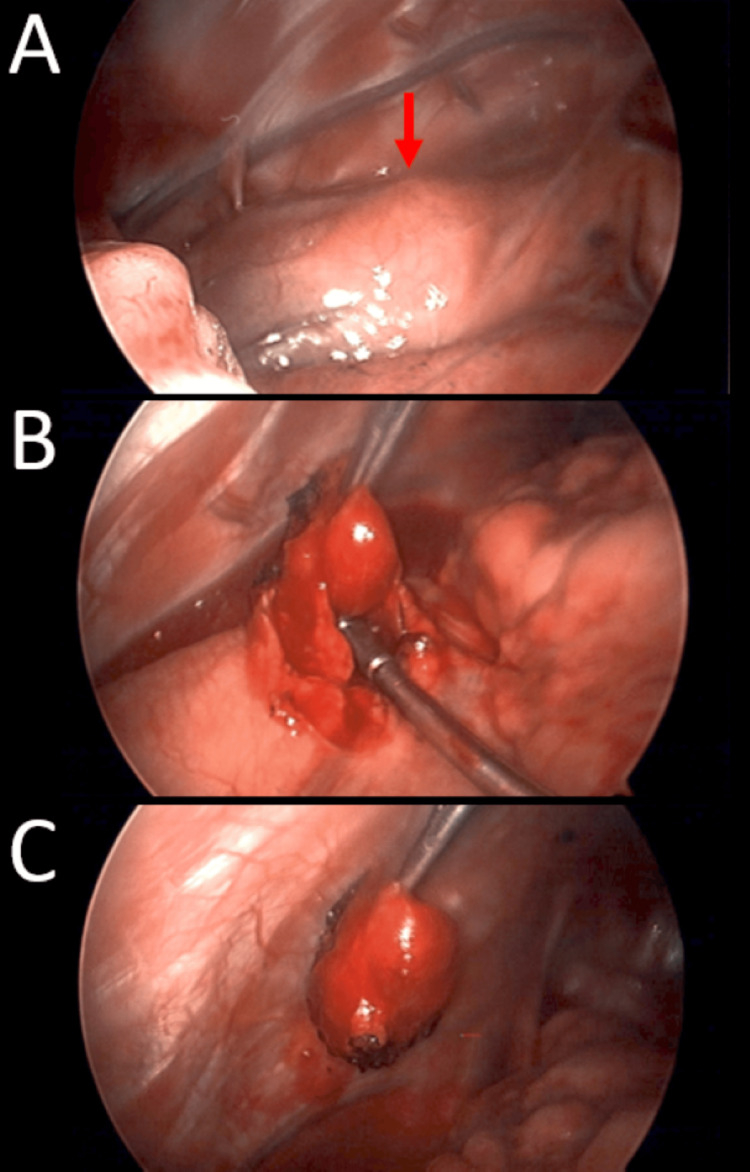
Intraoperative video-assisted thoracoscopic surgery (VATS) demonstrating excision of the causative lesion A rounded 1 cm mass was seen in the subpleural space, located below the aortic arch above the heart, and anterior to the phrenic nerve by several millimeters (red arrow, panel A). Electrocautery was lightly applied to the pleura, opening it, and dissection was very carefully performed with thoracic graspers. Gentle elevation with the suction tip was able to remove the lesion with minimal bleeding (panels B and C).

The patient underwent video-assisted thoracoscopic surgery (VATS) with successful identification and excision of the ectopic mediastinal parathyroid adenoma (Figure 3).

**Figure 4:**
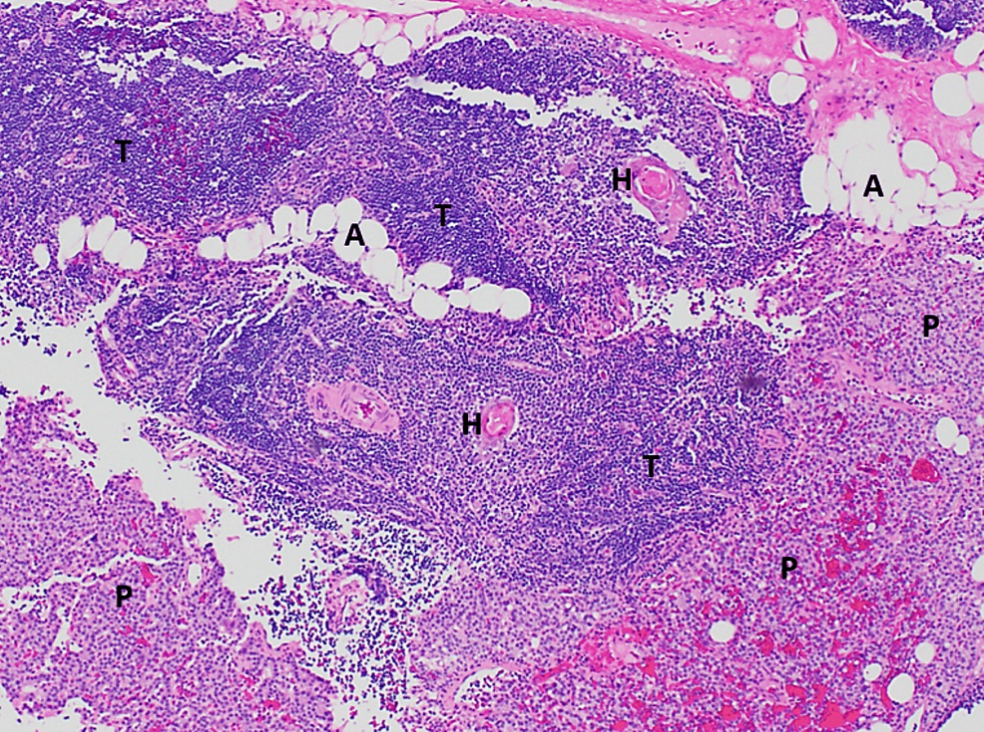
Microscopic surgical pathology of the excised lesion Low-magnification (40x) microphotograph demonstrating the parathyroid adenoma (lower part), which is located in the thymus (mid-upper part). Hematoxylin and eosin stain of the 2.4 cm x 1.5 cm x 0.5 cm surgically excised specimen, representing an intrathymic parathyroid adenoma. Microscopically, most of the parathyroid gland is surrounded by a capsule; however, in this focal area, the parathyroid glandular tissue (P) is mixed with thymus tissue (T). The thymus tissue contains Hassall corpuscles (H), and there are interspersed areas of adipose tissue (A). There is no evidence of increased mitosis, and these features are not typical of invasion; therefore, this represents an ectopic parathyroid gland within the thymus, which developed into a parathyroid adenoma.

Intact PTH levels were assessed and went from a preoperative level of >500 pg/mL to an intraoperative concentration of 77 pg/mL 10 minutes after the specimen was removed. Surgical pathology described the specimen as parathyroid glandular tissue surrounded by thymus and adipose tissue. The parathyroid component did not show evidence of increased mitosis, confirming the ectopic parathyroid specimen’s benign nature (Figure 4).

Postoperative and postdischarge sequela

The patient was discharged on postoperative day two with normal serum calcium levels. The patient made an excellent recovery with a complete resolution of her symptoms and normalization of her biochemistry profile.

## Discussion

Classical symptoms of hypercalcemia should prompt suspicion and evaluation for hyperparathyroidism. However, this patient presented to the emergency department with vague abdominal symptoms that do not necessarily elicit that unequivocal suspicion. Regardless, the typical work-up of virtually any patient presenting to the emergency department includes basic biochemical analysis. Following laboratory-determined hypercalcemia, a standard evaluation of intact serum PTH was conducted to determine if her hypercalcemia was from a PTH-dependent or PTH-independent process [7].

Elevated levels of serum intact PTH ascertained that her hypercalcemia was secondary to a PTH-dependent process. After ruling out multiple endocrine neoplasia syndromes (i.e., types 1 and 2a) that may also cause hyperparathyroidism [8,9], a parathyroid ultrasound (not shown) and nuclear medicine scan were negative (Figure 1A). Further exploration for an ectopic location with 99m Tc-sestamibi SPECT/CT demonstrated a focus of increased uptake in the prevascular mediastinal area (Figures 1B-1D). It has been previously described that this method of preoperative localization has improved the detection of ectopic glands [10-12]. A high-quality CT scan was used to analyze the prevascular anatomical region to aid in future surgical exploration. With the help of both imaging modalities, our team pursued a minimally invasive approach for this patient’s parathyroidectomy utilizing a left-sided approach with VATS. Although cases in the literature are rare, other authors have reported success using this diagnostic workflow of 99m Tc-sestamibi SPECT/CT scanning, surgical resection with a VATS approach, and pathological confirmation of parathyroid origin for mediastinally situated parathyroid adenomas [13,14]. Our experience presented here indicates that 99m Tc-sestamibi SPECT/CT scanning and VATS are appropriate options for the diagnosis, identification, and excision of an ectopic hyperparathyroidism-causing lesion in a young patient.

## Conclusions

The case reported here is among the few considering that ectopic parathyroid adenomas are rarely found within thymic tissue. Identification with advanced imaging modalities and excision with VATS are suitable diagnostic and treatment modalities in these situations of symptomatic hyperparathyroidism with secondary hypercalcemia.
